# RAD21 Confers Poor Prognosis and Affects Ovarian Cancer Sensitivity to Poly(ADP-Ribose)Polymerase Inhibitors Through DNA Damage Repair

**DOI:** 10.3389/fonc.2022.936550

**Published:** 2022-07-04

**Authors:** Rui Gou, Xiao Li, Hui Dong, Yuexin Hu, Ouxuan Liu, Juanjuan Liu, Bei Lin

**Affiliations:** ^1^ Department of Obstetrics and Gynecology, Shengjing Hospital of China Medical University, Shenyang, China; ^2^ Key Laboratory of Maternal-Fetal Medicine of Liaoning Province, Key Laboratory of Obstetrics and Gynecology of Higher Education of Liaoning Province, Shenyang, China

**Keywords:** RAD21, ovarian cancer, PARP inhibitor, DNA damage repair, prognosis

## Abstract

**Background:**

Poly(ADP-ribose)polymerase (PARP) inhibitors are a class of molecular-targeted cancer drugs. Synthetic lethality is a phenomenon that renders homologous recombination repair defective cells more sensitive to PARP inhibitors. As a component of the cohesin complex, RAD21 regulates DNA damage repair. However, the biological roles of RAD21 in ovarian cancer and their underlying mechanisms remain unclear.

**Methods:**

An immunohistochemical assay was used to validate the expression of RAD21 in ovarian cancer and its correlation with prognosis. The effects of RAD21 were evaluated through Cell Counting Kit-8 (CCK8), wound-healing, and invasion assays *in vitro* and the tumor growth *in vivo*. Furthermore, CCK8 assay and immunofluorescence assay were used to detect the effect of RAD21 on cell sensitivity to PARP inhibitors and their mechanism. The pathway changes were detected by Western blotting.

**Results:**

RAD21 was markedly upregulated in ovarian cancer samples. High RAD21 expression was correlated with poor differentiation and poor prognosis in patients with ovarian cancer. Functionally, RAD21 overexpression promoted cancer cell proliferation, migration, and invasion. Moreover, RAD21 knockdown increased the sensitivity of ovarian cancer cells to three kinds of PARP inhibitors by affecting DNA damage repair. *In vivo* experiments indicated that RAD21 promoted tumor growth. Mechanistically, the overexpression of RAD21 led to increased phosphorylation levels of Akt and mTOR. Blocking the Akt/mTOR signaling pathway reversed RAD21 overexpression-induced cancer progression and drug resistance.

**Conclusions:**

RAD21 can serve as a valuable prognostic marker for ovarian cancer and has the potential as a therapeutic target that can expand the utility of PARP inhibitors.

## Introduction

Ovarian cancer is a common female reproductive system malignant tumor. In 2020, 313,959 new ovarian cancer cases and 207,252 ovarian cancer-related deaths were reported worldwide (1). Currently, ovarian cancer treatment primarily relies on surgery and platinum-based chemotherapy (2). Despite the continuous improvement in surgical techniques and chemotherapy options, the long-term survival of patients with ovarian cancer remains unsatisfactory (3). Poly(ADP-ribose)polymerase (PARP) inhibitors are one of the targeted drugs recently approved for the maintenance therapy of ovarian cancer. PARP inhibitors cause DNA single-strand break repair defects and promote the formation of double-stranded breaks (DSBs) by targeting PARP. Cells with DSB repair defects cannot manage PARP inhibitor-induced DSB accumulation, eventually leading to cell death, a phenomenon known as synthetic lethality (4). However, the evaluation method for screening patients who are most likely to benefit from PARP inhibitor therapy needs to be improved, and drug combination approaches for homologous recombination (HR)-proficient patients need to be further evaluated.

*RAD21* codes for a human homolog of fission yeast *Saccharomyces pombe* Rad21 protein. SMC1, SMC3, RAD21, and SA1/2 form the cohesin complex, which is essential for various cellular processes (5). RAD21, a central component of the cohesin complex, regulates the sister chromatid cohesion and separation processes during mitosis, which are essential for proper chromosome segregation and DNA damage repair (6). RAD21 deficiency results in improper chromosome alignment in the metaphase and chromosome segregation defects in the anaphase. Affected cells have an HR-mediated repair defect, which leads to an increased level of spontaneous chromosomal breaks and ionizing radiation-induced chromosomal aberrations (7). Furthermore, RAD21 is involved in multiple biological functions, including gene transcription, centrosome biogenesis, cell apoptosis, meiosis, and hematopoiesis (5, 8). It has been reported that increased RAD21 expression was positively correlated with the progression of multiple cancers, such as breast cancer, cervical cancer, and colon cancer (9–11). However, the mechanisms underlying the role of RAD21 in ovarian cancer and its effect on the response to PARP inhibitors remain unknown, warranting further exploration.

Here, we explored the role of RAD21 in ovarian cancer *in vitro* and *in vivo*. We elucidated RAD21 expression in ovarian cancer and its correlation with patient prognosis by using a large number of clinical specimens. Based on cell experiments, we explored the function of RAD21 in ovarian cancer cell lines. Furthermore, we provided evidence for the effect of RAD21 on PARP inhibitor sensitivity and investigated the underlying molecular mechanism. Thus, our findings highlight RAD21 as a prognostic factor and suggest the efficacy of silencing RAD21 in combination with PARP inhibitors to treat ovarian cancer.

## Methods

### Patient Samples and Immunohistochemistry

A total of 130 paraffin-embedded specimens of ovarian tissue surgically removed from hospitalized patients from 2008 to 2012 at the Shengjing Hospital of China Medical University were selected. The patients had not undergone radiotherapy, chemotherapy, or hormone therapy before surgery. Pathologists examined the sections to achieve a diagnosis. The sections were divided into the following four groups: epithelial ovarian cancer (malignant tumor group, n = 95), epithelial ovarian borderline tumor (borderline tumor group, n = 15), epithelial ovarian benign tumor (benign tumor group, n = 11), and normal ovarian tissue (normal ovary group, n = 9). The study was approved by the Ethics Committee of China Medical University.

Paraffin-embedded ovarian tissue specimens were fixed in 10% formalin and then serially cut into 5-μm-thick sections. After being dewaxed, the samples were permeated with immunostaining permeation solution (Triton X-100, P0096, Beyotime, Shanghai, China), and the streptavidin–peroxidase connection method (UltraSensitive™ SP IHC Kit, KIT-9720, MXB, Fuzhou, China) was used to detect the expression of RAD21, following the manufacturer’s instructions. The concentration of the polyclonal antibody used against RAD21 was 1:250 (27071-1-AP; ProteinTech, Wuhan, China). The staining intensity was divided into not pigmented, light yellow, brownish-yellow, and dark brown and recorded as 0, 1, 2, and 3 points, respectively. The percentage of stained cells within the microscope field was classified into <5%, 5%–25%, 26%–50%, 51%–75%, and >75% and recorded as 0, 1, 2, 3, and 4 points, respectively. The final score was calculated by multiplying the two items: 0–2 points (−), 3–4 points (+), 5–8 points (++), and 9–12 points (+++). Two observers assessed the results independently to reduce errors.

### Cell Culture

Two human ovarian cancer cell lines (OVCAR3 and ES-2) were purchased from the Shanghai Cell Collection Center (Shanghai, China). The OVCAR3 cell line (adenocarcinoma) was cultured in Roswell Park Memorial Institute (RPMI) 1640 medium (BI) supplemented with 10% fetal bovine serum (FBS), whereas the ES-2 cell line (clear cell carcinoma) was cultured in McCoy’s 5A medium (BI) supplemented with 10% FBS. Both cell lines were incubated at 37°C with 5% CO_2_ and saturated humidity.

### Gene Transfection and Virus Infection

For the small interfering (si) RNA transfections, the cells were transfected with RAD21 siRNA using the liposome method (Lipo 3000 transfection kit, GIBCO, Invitrogen, Carlsbad, CA, USA). The RAD21 siRNA#1 (GenePharma, Shanghai, China) sequences were as follows: sense: 5′-GCAGCUUAUAAUGCCAUUATT-3′; antisense: 5′-UAAUGGCAUUAUAAGCUGCTT-3′. The RAD21 siRNA#2 sequences were as follows: sense: 5′-CCCAGCAGUUCAGCUUGAATT-3′; antisense: 5′-UUCAAGCUGAACUGCUGGGTT-3′. The control siRNA sequences were as follows: sense: 5′-UUCUCCGAACGUGUCACGUTT-3′; antisense: 5′-ACGUGACACGUUCGGAGAATT-3′. Stable RAD21 overexpression and control cell lines were established using GFP-expressing vectors according to the GeneChem (Shanghai, China) lentiviral gene transfection instructions, and the cells stably overexpressing RAD21 were screened using puromycin (Solarbio, Beijing, China) for at least 5 days.

### Western Blotting

Cells were harvested and lysed in pre-cooled radioimmunoprecipitation assay (RIPA) lysis buffer supplemented with protease inhibitors and phosphatase inhibitors. Total proteins were separated using sodium dodecyl sulfate–polyacrylamide gel electrophoresis (SDS-PAGE) gel and then transferred onto polyvinylidene difluoride (PVDF) membranes (Immobilon-P Transfer Membrane, Merck Millipore, Burlington, MA, USA). After the membrane was blocked with 5% non-fat milk for 2 h, the membrane was incubated with primary antibodies at 4°C overnight against RAD21 (1:1,000, 27071-1-AP, ProteinTech), matrix metalloproteinase 2 (MMP2) (1:1,000, 10373-2-AP, ProteinTech), MMP9 (1:1,000, 10375-2-AP, ProteinTech), mTOR (1:1,000, #2983, CST, Danvers, MA, USA), p-mTOR (1:1,000, #5536, CST), Akt (1:1,000, #4691, CST), p-Akt (1:1,000, #4060, CST), or GAPDH (1:1,500, TA-08, Zhongshan Jinqiao, Beijing, China). Subsequently, they were incubated with the horseradish peroxidase-labeled goat anti-rabbit IgG (H+L) (ZB-2301) or the horseradish peroxidase-labeled goat anti-mouse IgG (H+L) (ZB-2305, 1:5,000, Zhongshan Jinqiao) for 2 h at room temperature. The results were visualized using an enhanced chemiluminescence (ECL) reagent (Merck Millipore).

### Cell Counting Kit-8 Assay

Cells were seeded in 96-well plates at a density of 2,000 cells/well. After 6 h, the Cell Counting Kit-8 (CCK8) solution (B34304, Bimake, Houston, TX, USA) was added to cells at 0, 24, 48, 72, and 96 h and incubated for 2 h. The optical density values at 450 nm were measured using a universal microplate reader.

For drug treatment, cells were seeded in 96-well plates at a density of 5,000 cells/well. The next day, cells were treated with different concentrations of cisplatin (S1552), paclitaxel (SC0213), olaparib (SC9118), rucaparib (SC9145), and niraparib (SC6682, all purchased from Beyotime) and maintained in culture. After indicated days, the cells were incubated with CCK8 solution for 2 h. The optical density values at 450 nm were measured using a universal microplate reader.

### Wound-Healing Assay

A wound-healing assay was used to investigate cell migration. Cells were inoculated in a 6-well plate, and when cell confluence reached 90%, a 100-µl pipette tip was used to scratch the cell monolayer. Then, the cells were cultured in a serum-free culture medium for 24 h. Photographs of the wound were captured at 0 and 24 h after wounding using a camera-equipped microscope.

### Invasion Assay

Transwell chambers with Matrigel (BD Corporation, Franklin Lakes, NJ, USA) were used to measure cell invasion. Ovarian cancer cells (2 × 10^5^ cells/ml, 200 μl) diluted with the serum-free medium were added into the upper chamber, and 500 µl of medium containing 20% FBS was added to the lower chamber. After 48 h, the cells on the lower surface of the chamber were fixed with 4% paraformaldehyde for 30 min and stained with crystal violet for another 30 min. The number of stained cells was counted under a microscope.

### Immunofluorescence Assay

Cells were seeded on glass coverslips and cultured in the respective media containing PARP inhibitors for 4 days. Then, the cells were fixed in 4% paraformaldehyde for 20 min and permeabilized with Triton X-100 for 10 min at room temperature. After being blocked, the cells were incubated with the primary antibody against γ-H2AX (1:800, ab81299, Abcam, Cambridge, UK) at 4°C overnight, followed by incubation with CoraLite594-conjugated goat anti-rabbit IgG (H+L) (SA00013-4, ProteinTech) for 2 h at room temperature. The nuclei were stained using 4′,6-diamidino-2-phenylindole dihydrochloride for 5 min.

### Pathway Inhibition

Rapamycin complex kinase inhibitor Torkinib (PP242) was purchased from MedChemExpress (HY-10474, Monmouth Junction, NJ, USA). Cells were treated with 2 μmol/L of PP242 for 48 h and then collected for further experiments.

### Nude Mouse Xenograft Model

Twelve 4-week-old female BALB/cA-nu nude mice were purchased from Beijing Huafukang Biosciences (Beijing, China) and maintained in specific pathogen-free conditions. Approximately 5 × 10^6^ RAD21-overexpressing or control vector cells, suspended in 100 μl of PBS, were subcutaneously injected into the mouse axilla. The body weight and tumor volume were measured every 2 days. The tumor volume was calculated as follows: 0.5 × length × width × width. Finally, the mice were sacrificed, and the tumors were excised for the following detection analysis. The animal studies were approved by the Institutional Animal Research Committee of China Medical University.

### Database Analysis

The Human Protein Atlas (HPA) database (https://www.proteinatlas.org) was used to evaluate the expression levels of RAD21 and other proteins in ovarian cancer using immunohistochemical assays (12).

The Kaplan–Meier plotter (https://kmplot.com/analysis) was used to analyze the prognostic value of RAD21 in ovarian cancer (13). Log-rank *p* < 0.05 indicated statistically significant differences.

### Statistical Analyses

Statistical analyses were performed using the SPSS 22.0 software (IBM Corporation, Armonk, NY, USA). The count data were analyzed using the chi-square and Fisher’s exact probability tests. Statistical differences between two groups were analyzed using Student’s *t*-test, whereas multiple group differences were analyzed using the one-way ANOVA. The Kaplan–Meier and Cox regression models were used to analyze the independent risk factors and survival curve. *p* < 0.05 was considered statistically significant.

## Results

### RAD21 Was Upregulated in Epithelial Ovarian Cancers and Its Increased Expression Correlated With Poor Differentiation

**Figure 1:**
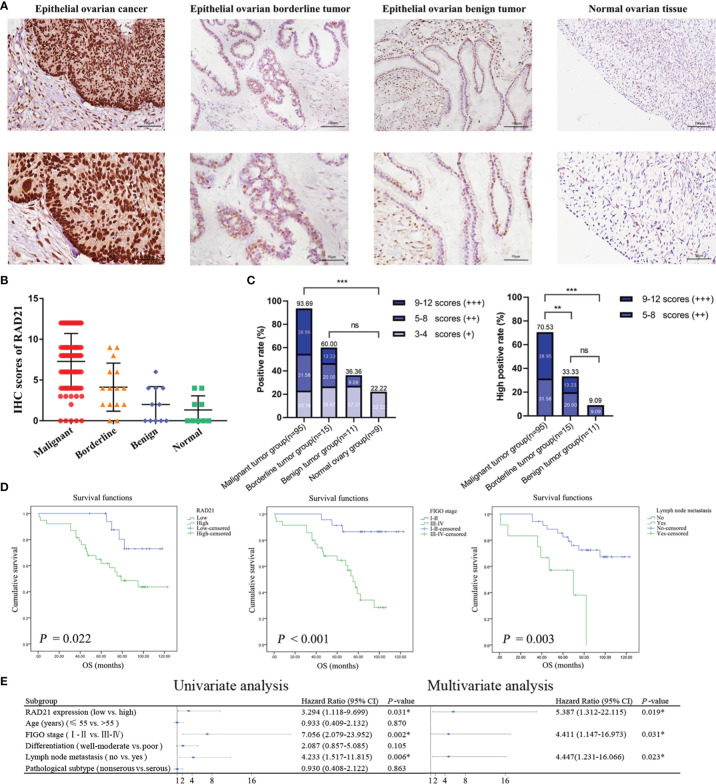
RAD21 expression level in different groups of ovarian tissue and its prognostic value in epithelial ovarian cancer. **(A)** Representative images of immunohistochemical staining for RAD21 in ovarian tissue samples. Magnification: upper, ×200; lower, ×400. **(B)** Immunostaining scores of RAD21 in ovarian tissue samples. **(C)** Statistical analyses of RAD21-positive and high-positive rates in ovarian tissue samples. **(D)** The relationship between RAD21 expression, International Federation of Gynecology and Obstetrics (FIGO) stage, lymph node metastasis, and overall survival in patients with epithelial ovarian cancer. **(E)** Forest plot based on Cox regression model analysis of patients with epithelial ovarian cancer. *p < 0.05; **p < 0.01;***p < 0.001; ns, not significant.

To verify the relationship between RAD21 and the occurrence and development of ovarian cancer, we detected its expression level in clinical specimens and analyzed its correlation with pathological parameters. Immunohistochemical assays demonstrated that RAD21 was mainly localized in the nucleus (**Figure 1A**). The positive and high-positive rates of RAD21 in the malignant tumor group (93.69% and 70.53%, respectively) were significantly higher than those in the borderline tumor group (60.00% and 33.33%, respectively), benign tumor group (36.36% and 9.09%, respectively), and normal ovary group (22.22% and 0.00%, respectively). No significant pairwise differences were observed between the other two groups (**Figures 1B, C**). HPA database analysis also confirmed that RAD21 stained moderately or intensely in ovarian cancer tissues and was mainly localized in the nucleus (**Supplementary Figure 1**).

A total of 95 patients with epithelial ovarian cancer were divided into high (++/+++) and low (−/+) RAD21 expression groups. Subsequently, we examined the clinicopathological characteristics of the patients in these groups. We found a significant correlation between RAD21 expression levels and degree of differentiation (*p* = 0.039), where patients with poor differentiation had a significantly higher percentage of RAD21 high positivity (78.57%) than those with well or moderate differentiation (58.97%). RAD21 showed the highest high-positive rate in ovarian endometrioid carcinoma (78.57%) and the lowest high-positive rate in ovarian mucinous carcinoma (57.14%). However, the RAD21 expression level was not significantly correlated with the International Federation of Gynecology and Obstetrics (FIGO) stage, lymph node metastasis, and pathological type (*p* > 0.05), as shown in **Table 1**.

**Table 1:** Relationship between RAD21 expression level in epithelial ovarian cancer and clinicopathological parameters.

Characteristics	n	Low			High		High positive rate (%)	*p*-Value
		(−)	(+)		(++)	(+++)		
**FIGO stage**								>0.05
I–II	38	4	10		11	13	63.16
III–IV	57	2	12		19	24	75.44
**Differentiation**								0.039*
Well – moderate	39	1	15		12	11	58.97
Poor	56	5	7		18	26	78.57
**LN metastasis**								
No	54	5	12		17	20	68.52	>0.05
Yes	22	0	5		9	8	77.27
No lymphadenectomy	19	1	5		4	9	68.42
**Pathologic type**								>0.05
Serous	45	2	11		18	14	71.11
Mucinous	7	0	3		1	3	57.14
Endometrioid	14	1	2		5	6	78.57
Clear cell	10	0	3		4	3	70.00
Poorly differentiated Adenocarcinoma	19	3	3		2	11	68.42

FIGO, International Federation of Gynecology and Obstetrics; LN, lymph node.

^*^p < 0.05.

### High RAD21 Expression Indicated Poor Prognosis

Ninety-five patients with epithelial ovarian cancer were followed up until April 30, 2019, and the Kaplan–Meier survival analysis results showed that the overall survival of patients with high RAD21 expression, FIGO III–IV, and lymph node metastasis was shorter than that of patients with low RAD21 expression, FIGO I–II, and no lymph node metastasis (**Figure 1D**). A Cox regression model was used to explore the relationship between clinicopathological parameters and prognosis. Univariate analysis suggested that high RAD21 expression, advanced FIGO stage, and lymph node metastasis were risk factors affecting the prognosis of patients with epithelial ovarian cancer. Incorporating the above three factors into the multivariate analysis, the results suggested that high expression of RAD21, advanced stage, and lymph node metastasis were independent risk factors affecting the survival and prognosis of patients with epithelial ovarian cancer (**Figure 1E**). Thus, RAD21 was upregulated in epithelial ovarian cancer and associated with a poor prognosis.

### RAD21 Promoted Ovarian Cancer Cell Proliferation, Migration, and Invasion

**Figure 2:**
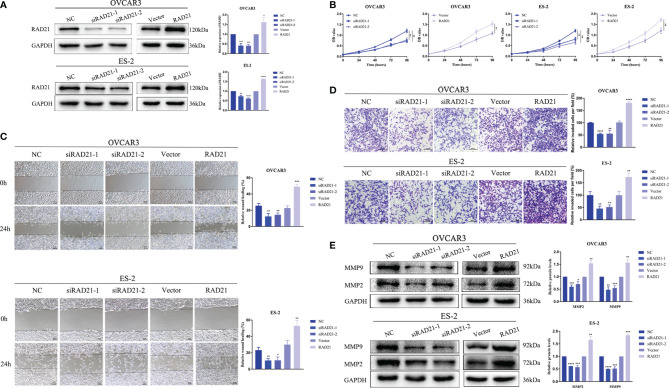
RAD21 overexpression promoted proliferation, migration, and invasion of ovarian cancer cells. **(A)** RAD21 expression after transfection with siRNA or lentivirus. **(B)** Ovarian cancer cell post-transfection proliferation ability evaluated using Cell Counting Kit-8 (CCK8) assays. **(C)** Ovarian cancer cell post-transfection migration ability evaluated using wound-healing assays. **(D)** Ovarian cancer cell post-transfection invasion ability evaluated using invasion assays. **(E)** The expression of matrix metalloproteinase 2 (MMP2) and MMP9 after transfection measured using Western blotting. Data were represented as mean ± SD (n = 3). *p < 0.05; **p < 0.01; ***p < 0.001; ****p < 0.0001.

We used siRNA to knock down RAD21 and lentivirus to overexpress RAD21 in the OVCAR3 and ES-2 cell lines (**Figure 2A**). CCK8, wound-healing, and invasion assays revealed that, compared with that in the control group, RAD21 knockdown significantly reduced cell proliferation, migration, and invasion in both cell lines, whereas RAD21 overexpression significantly enhanced cell proliferation, migration, and invasion (**Figures 2B–D**). Further results showed that RAD21 knockdown could significantly decrease the levels of MMP2 and MMP9, consistent with the characteristics of malignancy. Contrasting results were observed in cells with high RAD21 expression (**Figure 2E**).

### RAD21 Decreased the Antitumor Efficacy of Cisplatin and Poly(ADP-Ribose)Polymerase Inhibitors in Ovarian Cancer

Based on the role of RAD21 in DNA damage repair, we first evaluated the effect of RAD21 on the sensitivity of ovarian cancer cells to standard chemotherapeutics. After cells were treated with cisplatin or paclitaxel for 2 days, the CCK8 assay was conducted. Results showed that RAD21 knockdown increased the sensitivity of ovarian cancer cells to cisplatin but did not affect the effect of paclitaxel (**Supplementary Figures 2A–C**). We further used the Kaplan–Meier plotter to evaluate RAD21 in predicting the prognosis of ovarian cancer patients after chemotherapy. Results showed that ovarian cancer patients with high RAD21 expression had a poor prognosis after receiving platin (**Supplementary Figure 2D**). However, RAD21 could not predict the prognosis of receiving taxol (**Supplementary Figure 2E**).

**Figure 3:**
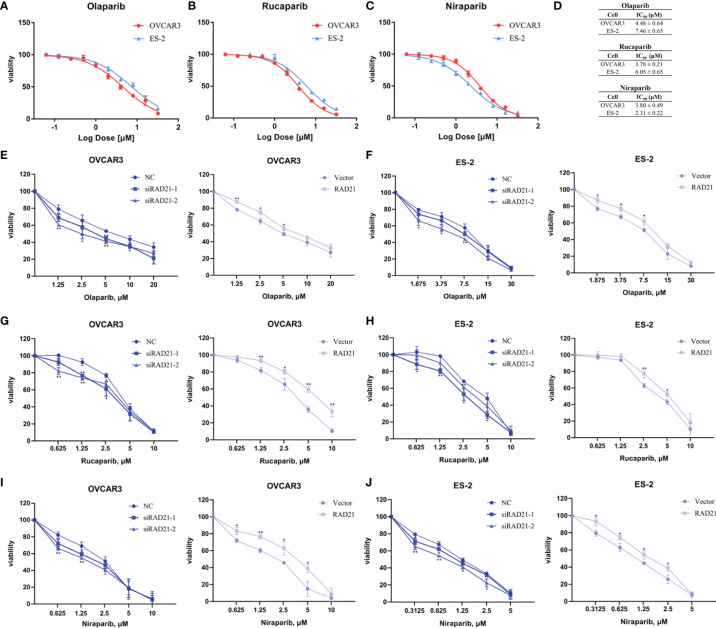
RAD21 overexpression reduced poly(ADP-ribose)polymerase (PARP) inhibitor sensitivity in ovarian cancer cells. **(A–C)** Cell viability in cells treated with olaparib **(A)**, rucaparib **(B)**, and niraparib **(C)** analyzed using the Cell Counting Kit-8 (CCK8) assay. **(D)** IC_50_ values in cells treated with PARP inhibitors. **(E, F)** The effects of RAD21 on olaparib treated OVCAR3 **(E)** and ES-2 **(F)** cell proliferation. **(G, H)** The effects of RAD21 on rucaparib-treated OVCAR3 **(G)** and ES-2 **(H)** cell proliferation. **(I, J)** The effects of RAD21 on niraparib-treated OVCAR3 **(I)** and ES-2 **(J)** cell proliferation. Data were represented as mean ± SD (n = 3). *p < 0.05; **p < 0.01.

PARP inhibitors have achieved satisfactory results as targeted therapy drugs. Currently, olaparib, rucaparib, and niraparib have been approved by the Food and Drug Administration (FDA) for the maintenance therapy of ovarian cancer. After OVCAR3 and ES-2 cells were treated with these three drugs for 4 days, the CCK8 assay was conducted. Results showed that the cell viability decreased in a dose-dependent manner (**Figures 3A–C**). The OVCAR3 cell line was more sensitive to olaparib and rucaparib, whereas the ES-2 cell line was more sensitive to niraparib (**Figure 3D**).

To determine the effect of RAD21 on ovarian cancer cell proliferation when treated with PARP inhibitors, cells were transfected with siRNA or lentivirus and treated with the indicated concentration of PARP inhibitors for 4 days. The CCK8 assay was used to confirm the sensitivity of ovarian cancer cells to PARP inhibitors. Results showed that the RAD21 siRNA-treated OVCAR3 and ES-2 cell lines were more sensitive to olaparib, rucaparib, and niraparib than those in the control groups. Conversely, cells with high RAD21 expression gained resistance to these PARP inhibitors (**Figures 3E–J**, **Supplementary Table 1**). Therefore, the upregulation of RAD21 expression attenuated the inhibitory effect of PARP inhibitors on OVCAR3 and ES-2 cell proliferation.

### RAD21 Affected Double-Stranded Break Repair in Poly(ADP-Ribose)Polymerase Inhibitor-Treated Cells

**Figure 4:**
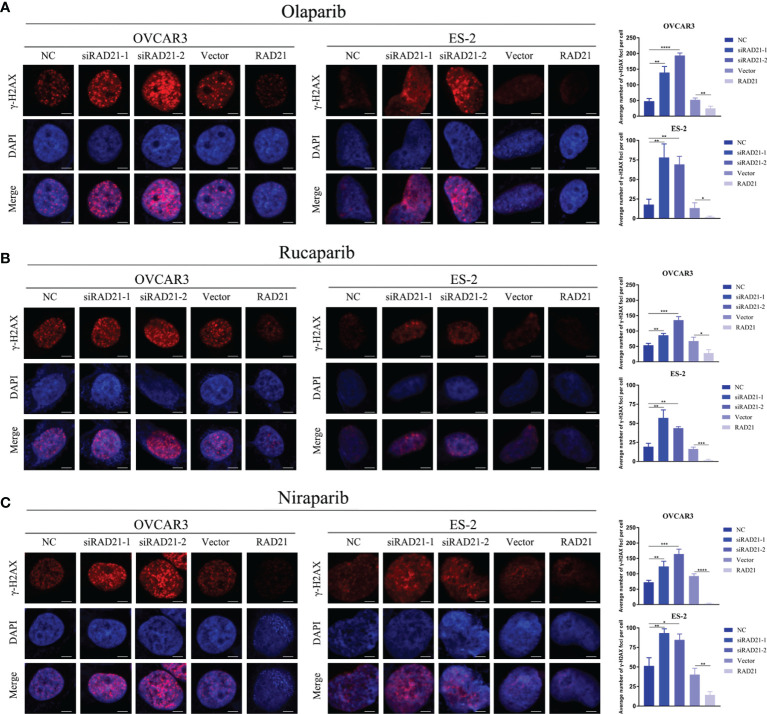
Effects of RAD21 on DSB repair after treatment with poly(ADP-ribose)polymerase (PARP) inhibitors. **(A–C)** Representative images of immunofluorescence staining for γ-H2AX and numerical quantification of γ-H2AX foci in ovarian cancer cells treated with olaparib **(A)**, rucaparib **(B)**, and niraparib **(C)**. Scale bar = 10 μm. Data were represented as mean ± SD (n = 3). *p < 0.05; **p < 0.01; ***p < 0.001; ****p < 0.0001.

To evaluate whether the knockdown of RAD21 sensitized ovarian cancer cells to PARP inhibitors by impairing DNA damage repair, we treated siRNA- or lentivirus-transfected cells with PARP inhibitors for 4 days at IC_50_ concentrations and quantified DSB formation based on the presence of γ-H2AX foci. The immunofluorescence assay results indicated that, compared with that in the control group, knockdown of RAD21 combined with treatment with three PARP inhibitors led to an increase in the formation of γ-H2AX foci, whereas overexpressing RAD21 combined with PARP inhibitor treatment significantly reduced the formation of γ-H2AX foci, which was consistent with the results of the cell viability assay (**Figures 4A–C**). Therefore, RAD21 may reduce the sensitivity of ovarian cancer cells to PARP inhibitors by facilitating DSB repair.

### RAD21 Promoted Ovarian Cancer Progression by Activating the Akt/mTOR Signaling Pathway

**Figure 5:**
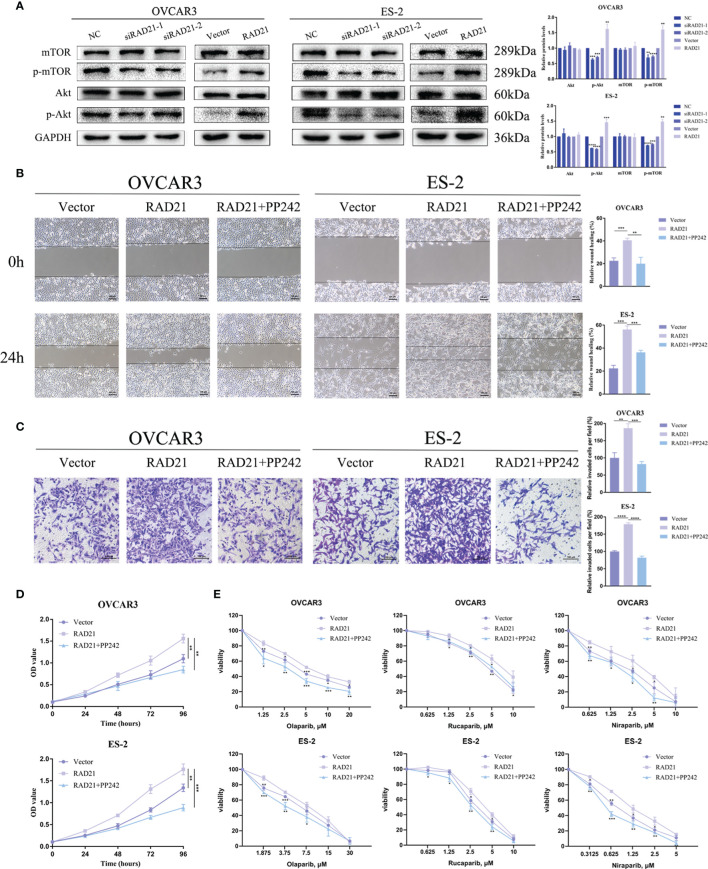
RAD21 regulated ovarian cancer progression by activating the Akt/mTOR signaling pathway. **(A)** Western blotting analysis showing the levels of Akt, p-Akt, mTOR, and p-mTOR in ovarian cancer cells with RAD21 knockdown or overexpression. **(B)** Wound-healing assay showing the migration ability of ovarian cancer cells treated with PP242. **(C)** Invasion assay showing the invasion ability of ovarian cancer cells treated with PP242. **(D)** Cell Counting Kit-8 (CCK8) assay showing the proliferation ability of ovarian cancer cells treated with PP242. **(E)** Cell viability of ovarian cancer cells treated with PP242 and poly(ADP-ribose)polymerase (PARP) inhibitors. Data were represented as mean ± SD (n = 3). *p < 0.05; **p < 0.01; ***p < 0.001; ****p < 0.0001.

To investigate the potential mechanism underlying the promotion of ovarian cancer progression by RAD21, we investigated the signaling pathways related to RAD21 using Western blotting. The results from OVCAR3 and ES-2 cells demonstrated that silencing RAD21 led to decreased levels of p-Akt and p-mTOR, whereas RAD21 overexpression led to the opposite results (**Figure 5A**). To explore whether RAD21 promoted malignant biological behaviors in ovarian cancer cells *via* the Akt/mTOR signaling pathway, we performed rescue experiments. We found that the increased cell migration, invasion, and proliferation caused by RAD21 upregulation could be abrogated by the mTOR inhibitor PP242 in OVCAR3 and ES-2 cells (**Figures 5B–D**). Furthermore, PP242 reversed the RAD21 upregulation-induced resistance to PARP inhibitors in ovarian cancer cells (**Figure 5E**). Thus, RAD21 promoted ovarian cancer progression by activating the Akt/mTOR signaling pathway.

### RAD21 Promoted Tumor Growth *In Vivo*

**Figure 6:**
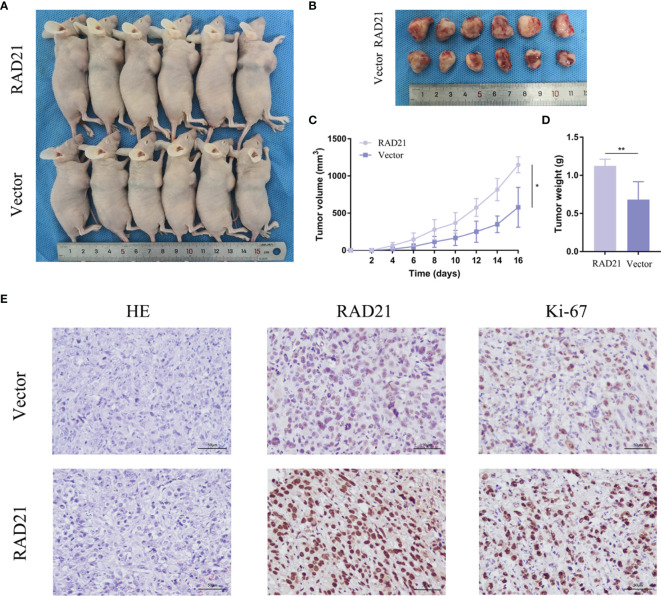
The impact of RAD21 on tumor formation *in vivo*. **(A)** Subcutaneous xenograft nude mouse model. **(B)** Images of tumors from nude mice in the vector and RAD21-overexpressing groups. **(C)** Subcutaneous tumor volume in the two groups. **(D)** Tumor weight in the two groups. **(E)** H&E and immunohistochemical staining in the two groups. *p < 0.05; **p < 0.01.

**Figure 7:**
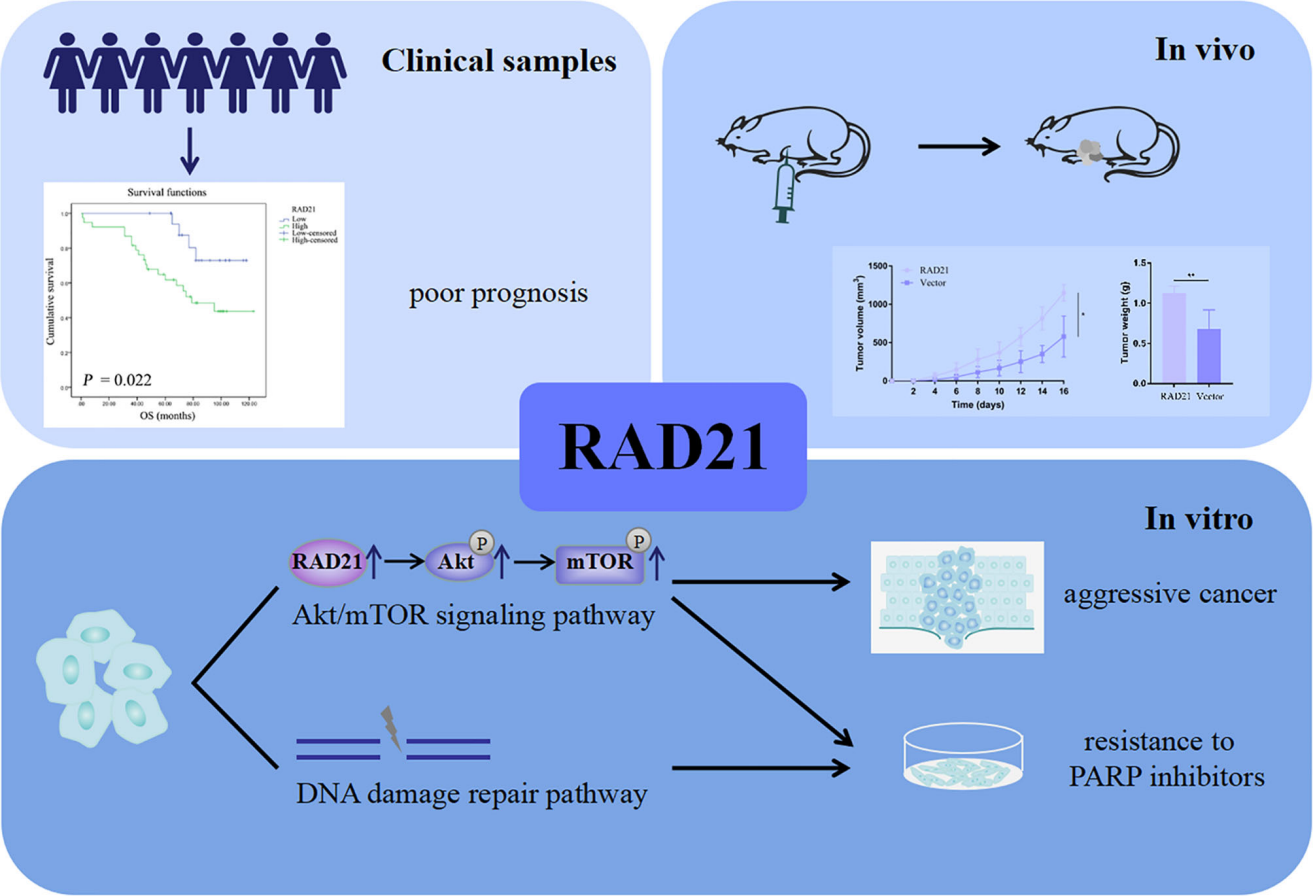
A schematic diagram illustrating the role of RAD21 in ovarian cancer.

Nude mice were subcutaneously injected with ES-2 vector cells and ES-2 RAD21 overexpression cells. Changes in RAD21 expression in these cells positively affected the volume and weight of subcutaneous tumors (**Figures 6A–D**). Ki-67 protein exists in all active stages of the cell cycle, and its expression level correlates with tumor cell proliferation activity. The immunohistochemical results showed that the Ki-67 staining of the RAD21 overexpression group was darker than that of the control group, indicating the pro-proliferative role of RAD21 in ovarian cancer (**Figure 6E**). Collectively, these results indicated that RAD21 plays an important role in ovarian cancer progression (**Figure 7**).

## Discussion

Accurate DNA damage repair is critical to genome integrity and cell survival. DNA damage repair defects cause increased genomic instability, leading to the heterogeneity and other malignant features of ovarian cancer (14). DSBs are considered the most cytotoxic DNA lesions (15). HR, an error-free repair mechanism, is the main DSB repair method (4). Based on the synthetic lethal effect, PARP inhibitors exhibited promising antitumor efficacy in ovarian cancer patients with HR deficiency (16). However, patients with *de novo* or acquired HR proficiency could not benefit from PARP inhibitors (17). Therefore, identifying the combination therapy strategy that effectively enhances the antitumor activity of PARP inhibitors has become a new research direction. As an important protein involved in the process of HR repair, RAD21 has attracted our attention. Here, we demonstrated that RAD21 was highly expressed in ovarian cancer, and its expression correlated with poor prognosis. RAD21 promoted malignant biological behaviors of ovarian cancer cells by activating the Akt/mTOR signaling pathway and reduced the sensitivity of cancer cells to PARP inhibitors by affecting the DSB repair. Therefore, RAD21 plays a crucial role in ovarian cancer progression and is expected to become a prognostic factor and a therapeutic target for combination therapy with PARP inhibitors.

Cohesin, an essential multiprotein complex, undergoes loading and unloading onto chromosomes in a dynamic fashion during the cell cycle, mediating sister chromatid cohesion and ensuring the equal separation of the genetic material to daughter cells (18, 19). When DNA damage was created at discrete sites in the cell nucleus using a laser microbeam, cohesin was recruited to the damaged region in a Mre11/Rad50-dependent manner (20). Cohesin facilitates sister chromatid HR repair by mediating the cohesion between the broken chromatid and its intact sister template (21). In addition, cohesin prevents the end joining of distant DSB ends to prevent deleterious genome rearrangements (22). Furthermore, cohesin accumulation facilitates the recruitment of checkpoint proteins, activating the intra-S and G2/M checkpoints (23).

Cohesin dysregulation leads to developmental disease and cancer. Unlike other subunits that are prone to mutation, *RAD21* is most frequently amplified in cancers (24). Enhanced RAD21 expression was correlated with early relapse and chemotherapy resistance in high-grade luminal, basal, and HER2 breast cancers (9). RAD21 was significantly overexpressed in cervical intraepithelial neoplasia III and cervical cancer and involved in the regulation of cell cycle and RNA transportation, promoting cervical cancer progression (10). RAD21 expression in colorectal cancer cells was modulated by Wnt/β-catenin activation and was associated with shorter disease-specific survival in patients with *KRAS* mutant tumors (11, 25). Furthermore, high RAD21 expression conferred poor survival in patients with non-small cell lung cancer, especially in stages II–III (26). However, the correlation between RAD21 and ovarian cancer prognosis has not been clarified. We found that RAD21 was overexpressed in ovarian cancer and that patients with high RAD21 expression showed poor differentiation and poor overall survival. Furthermore, RAD21 overexpression increased ovarian cancer cell migration and invasion. Moreover, the MMP2 and MMP9 expression levels were consistent with those of RAD21. Studies showed that MMPs participated in every step from tumorigenesis to metastatic implantation in ovarian cancer by degrading various component proteins in the extracellular matrix, such as collagen and casein (27). Among them, MMP9 regulates cell–cell adhesion by catalyzing E-cadherin ectodomain shedding to mediate metastatic dissemination, whereas MMP2 mediates the peritoneal adhesion of ovarian cancer cells by cleaving the extracellular matrix proteins fibronectin and vitronectin into small fragments (28, 29). Therefore, RAD21 likely promoted the progression of ovarian cancer by regulating the expression of MMP2 and MMP9, and its high expression predicted poor prognosis of ovarian cancer patients.

As a protein that plays an important role in DSB repair, RAD21 has been reported to be associated with cancer therapy resistance. A study showed that *Rad21* heterozygous mutant cells were defective in DNA damage checkpoint activation and DSB repair. *Rad21^+/−^* mice were more sensitive to whole-body irradiation (30). RAD21 expression was upregulated within ionizing radiation-insensitive hepatocellular carcinoma tissues, which led to reduced cell sensitivity to therapy by attenuating ionizing radiation treatment-induced DNA damage (31). In breast cancer, *RAD21* was identified as a gene related to tamoxifen treatment response (32, 33). Furthermore, suppression of RAD21 expression enhanced the cytotoxicity of the two DNA-damaging chemotherapy drugs etoposide and bleomycin (34). In non-small cell lung cancer, high expression of RAD21 mediated cisplatin resistance by enhancing DNA damage repair (35). Surprisingly, a recent case report demonstrated that a patient with metastatic intrahepatic cholangiocarcinoma carrying a BRCA1-associated protein 1 (*BAP1*) mutation and *RAD21* amplification benefited from PARP inhibitor treatment (36). BAP1, a ubiquitin carboxy-terminal hydrolase, interacts with the RING finger domain of BARD1 to inhibit the E3 ligase activity of BRCA1/BARD1, protecting BRCA1 from proteasome-mediated degradation (37, 38). Therefore, deficient HR repair caused by a *BAP1* mutation rather than *RAD21* dysregulation may explain why this patient benefited from PARP inhibitor treatment. Currently, no other studies have explored the effect of RAD21 on the therapeutic efficacy of PARP inhibitors. We found that high expression of RAD21 increased the resistance of ovarian cancer cells to three PARP inhibitor types, and PARP inhibitor-induced cytotoxicity was augmented by inhibiting RAD21 expression. This suggests a synthetic lethality relationship between PARP inhibitors and RAD21 deficiency. When DSBs are generated, H2AX is rapidly phosphorylated at serine 139. Phosphorylated H2AX is the initial step in the recruitment and localization of DNA repair proteins, and the accumulation of γ-H2AX foci is considered a sensitive biomarker of DSB damage (39). We found that high RAD21 expression significantly reduced the accumulation of PARP inhibitor-induced γ-H2AX foci, suggesting that RAD21 affected the response of ovarian cancer cells to PARP inhibitors by participating in the DNA damage repair. PARP inhibitor treatment leads to the accumulation of DNA single-strand breaks and stalling of replication forks, which further aggregates DSBs (40). RAD21, by exerting a DSB repair function, enables rapid repair of DNA breaks caused by PARP inhibitor treatment, leading to cell survival and thus the resistance of ovarian cancer cells to PARP inhibitors. Therefore, patients with low RAD21 expression are likely to benefit from PARP inhibitors. A combination of inhibiting the RAD21-mediated DNA damage repair pathway with PARP inhibitors may be a new therapeutic strategy for HR-proficient cancers.

To explore the mechanism, we tested a variety of signaling pathways and found that RAD21 promoted Akt and mTOR phosphorylation. Emerging evidence suggests that the Akt/mTOR pathway not only participates in anti-apoptosis, angiogenesis, aggressive phenotype, and platinum resistance but also plays a role in DNA damage repair (41, 42). In soft tissue sarcomas and gastrointestinal stromal tumors, Akt signaling inhibition decreased the RAD51 protein level by regulating protein stability, decreasing the efficacy of homology-mediated repair of DNA DSBs, and ultimately sensitizing tumor cells to doxorubicin (43). Combining with a PI3K/mTOR pathway inhibitor mitigated the CHK1 inhibitor-induced RAD51-mediated HR repair and augmented replication stress, leading to cell death in high-grade serous ovarian carcinoma (44). Recently, studies showed that mTOR inhibition promoted adaptive mutagenesis by impairing accurate DNA repair and that the selective mTOR inhibitor PP242 delayed the repair of ionizing radiation-induced DNA DSBs (45). Furthermore, inhibiting mTORC1/2 using PP242 induced the formation of γ-H2AX foci in inflammatory breast cancer cells (46). When we treated RAD21-overexpressing ovarian cancer cells with PP242, the high ability of cell proliferation, migration, and invasion induced by RAD21 overexpression was reduced. Furthermore, PP242 treatment attenuated the resistance of RAD21-overexpressing ovarian cancer cells to PARP inhibitors. Considering the role of the Akt/mTOR pathway in DNA damage repair, we hypothesize that RAD21 affects the ovarian cancer cell response to PARP inhibitors not only by mediating sister chromatid cohesion but also by activating the Akt/mTOR pathway.

In summary, we revealed that RAD21 conferred a poor prognosis in ovarian cancer patients. RAD21 played a key role in ovarian cancer progression and reduced sensitivity to PARP inhibitors through DNA damage repair. Thus, RAD21 could serve as a novel prognostic ovarian cancer biomarker and a predictive target for the therapeutic efficacy of PARP inhibitors. Targeting RAD21 may expand the number of patients benefiting from PARP inhibitors.

## Data Availability Statement

The original contributions presented in the study are included in the article. Further inquiries can be directed to the corresponding author.

## Ethics Statement

The studies involving human participants were reviewed and approved by the Ethics Committee of China Medical University, and an informed consent exemption was approved (Project ID: 2022PS415K). Written informed consent for participation was not required for this study in accordance with the national legislation and the institutional requirements. The animal study was reviewed and approved by the Institutional Animal Research Committee of China Medical University (Project ID: 2022PS350K).

## Author Contributions

RG and XL conceived the study. RG performed the experiments and wrote the manuscript. HD, YH, and OL contributed to the sample collection. JL contributed to the data collection. BL revised the manuscript. All authors read and approved the final manuscript.

## Funding

This study was approved by the Beijing Kanghua Foundation for the Development of Traditional Chinese and Western Medicine Gynecological Oncology Special Research Fund (KH-2021-LLZX-010), the Key R&D Guidance Plan Project in Liaoning Province (2019JH8/10300022), and the National Natural Science Foundation of China (82173130).

## Conflict of Interest

The authors declare that the research was conducted in the absence of any commercial or financial relationships that could be construed as a potential conflict of interest.

## Publisher’s Note

All claims expressed in this article are solely those of the authors and do not necessarily represent those of their affiliated organizations, or those of the publisher, the editors and the reviewers. Any product that may be evaluated in this article, or claim that may be made by its manufacturer, is not guaranteed or endorsed by the publisher.
