# Supplementary material for: RAD21 Confers Poor Prognosis and Affects Ovarian Cancer Sensitivity to Poly(ADP-Ribose)Polymerase Inhibitors Through DNA Damage Repair
Source: Front Oncol. 2022 Jul 4;12:936550. doi: 10.3389/fonc.2022.936550 (PMC9289200; doi:10.3389/fonc.2022.936550)
Supplement: Supplementary file 1 [file DataSheet_1.pdf]

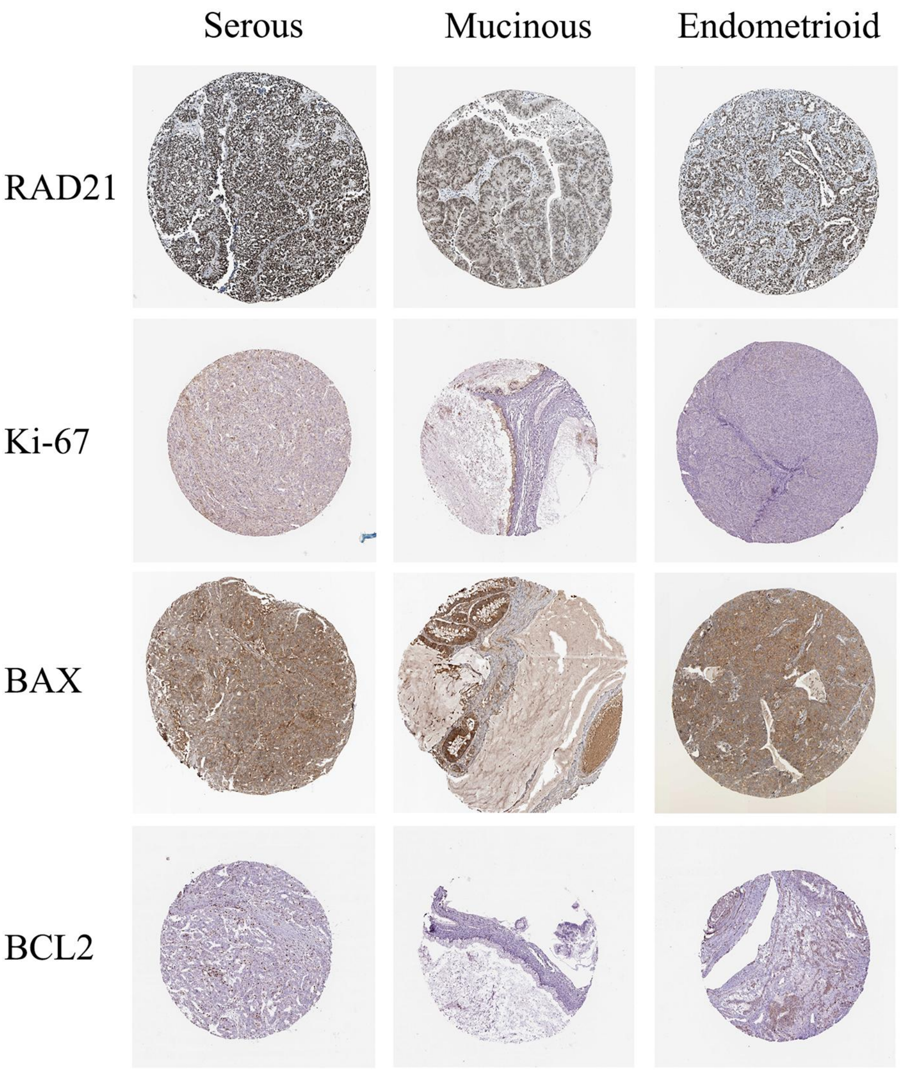


**Supplementary figure 1.** Protein expression levels of RAD21, Ki-67, BAX, and BCL2 in ovarian cancer (HPA dabatase).

A B

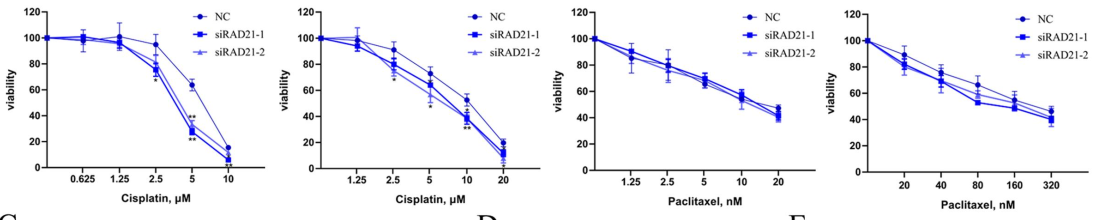


C

| Cell | Group | IC$_{50}$ (μM) | IC$_{50}$ (nM) |
|---|---|---|---|
| | | Cisplatin | Paclitaxel |
| OVCAR3 | NC | 5.95 ± 0.52 | 14.11 ± 3.41 |
| | siRAD21-1 | 3.65 ± 0.14 | 13.30 ± 2.24 |
| | siRAD21-2 | 4.06 ± 0.23 | 11.91 ± 3.27 |
| ES-2 | NC | 9.71 ± 0.88 | 219.70 ± 79.40 |
| | siRAD21-1 | 6.93 ± 0.57 | 141.60 ± 45.70 |
| | siRAD21-2 | 6.23 ± 0.95 | 169.60 ± 75.00 |

D E

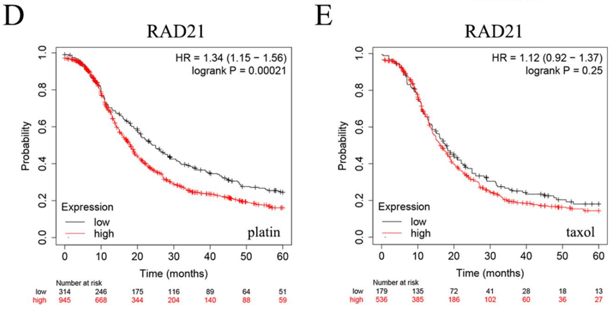


**Supplementary figure 2.** RAD21 affected cisplatin sensitivity in ovarian cancer. (A) The effects of RAD21 on cisplatin treated cell proliferation. (B) The effects of RAD21 on paclitaxel treated cell proliferation. (C) IC$_{50}$ values in cells treated with cisplatin or paclitaxel. (D) Prognostic significance of RAD21 in patients with ovarian cancer treated with platin. (E) Prognostic significance of RAD21 in patients with ovarian cancer treated with taxol.
