# Supplementary material for: RAD21 Confers Poor Prognosis and Affects Ovarian Cancer Sensitivity to Poly(ADP-Ribose)Polymerase Inhibitors Through DNA Damage Repair
Source: Front Oncol. 2022 Jul 4;12:936550. doi: 10.3389/fonc.2022.936550 (PMC9289200; doi:10.3389/fonc.2022.936550)
Supplement: Supplementary file 2 [file Table_1.doc]

**Supplementary table 1** The IC_50_ values of PARP inhibitors in different groups.

| **Cell** | **Group** | IC_50_ (μM) | | |
| --- | --- | --- | --- | --- |
|  |  | **Olaparib** | **Rucaparib** | **Niraparib** |
| OVCAR3 | NC | 7.10 ± 1.98 | 4.09 ± 0.21 | 2.20 ± 0.35 |
|  | siRAD21-1 | 4.28 ± 1.17 | 3.07 ± 0.33 | 1.79 ± 0.36 |
|  | siRAD21-2 | 3.71 ± 1.54 | 3.18 ± 0.51 | 1.55 ± 0.31 |
|  | Vector | 5.72 ± 1.16 | 3.43 ± 0.28 | 1.75 ± 0.31 |
|  | RAD21 | 8.14 ± 1.30 | 6.40 ± 0.46 | 3.20 ± 0.54 |
| ES-2 | NC | 7.74 ± 1.18 | 4.22 ± 0.53 | 1.20 ± 0.15 |
|  | siRAD21-1 | 6.72 ± 1.17 | 2.74 ± 0.26 | 1.02 ± 0.19 |
|  | siRAD21-2 | 4.99 ± 1.01 | 3.55 ± 0.41 | 0.79 ± 0.16 |
|  | Vector | 6.57 ± 0.92 | 3.83 ± 0.39 | 1.01 ± 0.12 |
|  | RAD21 | 9.25 ± 0.85 | 5.04 ± 0.50 | 1.49 ± 0.19 |
